# Unraveling complex interactions of meteorological factors and sulfur dioxide on other infectious diarrhea: evidence from a subtropical city

**DOI:** 10.3389/fpubh.2026.1706739

**Published:** 2026-04-13

**Authors:** Jiangwang Fang, Xiaoyan Zheng, Hanwei Wang, Danjing Chen, Zhiying Zhan, Xian-E Peng

**Affiliations:** 1 Department of Epidemiology and Health Statistics, Fujian Provincial Key Laboratory of Environment factors and Cancer, School of Public Health, Fujian Medical University, Fuzhou, Fujian Province, China; 2 Emergency Response Department, The Affiliated Fuzhou Center for Disease Control and Prevention of Fujian Medical University, Fuzhou, Fujian Province, China; 3 Department of Infectious Disease Prevention and Control, The Affiliated Fuzhou Center for Disease Control and Prevention of Fujian Medical University, Fuzhou, Fujian Province, China

**Keywords:** mean relative humidity (MRH), mean temperature (MT), other infectious diarrhea, sulfur dioxide (SO_2_), the interaction effect

## Abstract

**Background:**

The incidence of other infectious diarrhea (OID) is rising, imposing a significant medical burden. While meteorological factors affect OID, few studies have examined sulfur dioxide (SO_2_) or its interactions with these variables.

**Methods:**

Using quasi-Poisson and distributed lag non-linear models, we analyzed the lagged, non-linear effects of meteorological factors and SO_2_ on OID cases in Fuzhou, China (2015–2019). Interactive effects between SO_2_ and meteorological variables were evaluated using multiplicative terms, multiplicative scale, relative excess risk due to interaction (RERI), and attributable proportion (AP). Stratified analyses highlighted vulnerable groups.

**Results:**

The relationship between OID and mean temperature (MT), mean relative humidity (MRH) and SO_2_ were represented by U-, L-, and J-shaped curves, respectively. A synergistic effect was found between MT and MRH, where low levels of both increased OID risk, while SO_2_ and MT showed an antagonistic effect, with high SO_2_ and low MT raising OID risk. Children aged 3–4 years were particularly sensitive to extremely low MT.

**Conclusion:**

These results underscore the importance roles of MT, MRH, and SO_2_ in OID risk, and highlight the need for prediction and prevention strategies targeting the co-exposure of environmental factors.

## 1 Introduction

Infectious diarrhea (ID) is a common global disease and a key indicator of regional health, food safety, and public health, particularly causing malnutrition and mortality in children under five in developing countries (1). In China, other infectious diarrhea (OID) excludes cholera, dysentery, typhoid/paratyphoid, and is classified as a category C notifiable disease (2). OID incidence remains high, consistently ranking among the top three class C diseases annually, with an average annual incidence of 60.64/100,000 from 2004–2017, showing an increasing trend [annual percentage change (APC) = 4.12] (3). OID cases peak in summer (linked to Escherichia coli and Salmonella) and winter (linked to rotavirus and norovirus) (4), posing significant public health and economic challenges, particularly for young children (3). Enhanced efforts are needed for OID prevention and control.

Climate change is a major environmental challenge, with meteorological factors linked to both chronic and infectious diseases (5). Temperature impacts infectious diseases with varying lagged effects: 1–2 weeks for influenza, 3–6 weeks for diarrhea, and 7–12 weeks for malaria. Meteorological factors influence the persistence and infectivity of bacteria (6) and viruses (7), contributing to OID. Studies have shown that temperature (8) and humidity (9) significantly affect infectious diarrhea occurrence. For instance, research on 270 Chinese cities (4) found a non-linear relationship between temperature and OID transmission, while lower relative humidity in Guangzhou (8) was linked to higher ID risk, varying with lag time. Although interactions between meteorological factors and infectious diseases like influenza (10) and tuberculosis (11) are known, evidence on their interactive effects on OID remains limited (12).

Air pollution is another pressing environmental issue, with sulfur dioxide (SO_2_) being a major atmospheric pollutant. SO_2_ can form secondary pollutants like acids and sulfates (13), posing risks to respiratory diseases (14) and type 2 diabetes (15). A review article (16) on the association between air pollutants and gastrointestinal diseases suggests that exposure to air pollutants such as particulate matter with aerodynamic diameter ≤ 2.5 micrometers (PM_2.5_), particulate matter with aerodynamic diameter ≤ 10 micrometers (PM_10_), nitrogen dioxide (NO_2_), sulfur dioxide (SO_2_) and ozone (O_3_) were either positively or negatively associated with various intestinal disorders. Notably, the detrimental effects of SO_2_ on gastrointestinal diseases showed a relatively consistent pattern across studies. Acute exposure to SO_2_ has been linked to Helicobacter pylori infections (17), but its effects on intestinal diseases, including OID, remain largely unexplored. A study conducted in Hangzhou, China, reported that higher concentrations of SO_2_ were associated with norovirus infection among children (18). Given that norovirus is one of the most common pathogens responsible for OID, this finding provides indirect evidence supporting a potential link between SO_2_ exposure and OID incidence. Given the regional variations in OID prevalence and environmental influences, more studies in tropical and subtropical regions with high temperature and humidity are urgently needed.

Environmental exposures encompass both meteorological factors and air pollutants, and virtually all individuals are concurrently exposed to these factors in real-world settings. Previous studies have shown that meteorological factors and air pollutants interact in influencing the incidence of infectious diseases such as tuberculosis (19), influenza (20), and hand, foot, and mouth disease (21). Therefore, it is essential to consider the combined effects of meteorological factors and air pollutants when evaluating their impacts on health outcomes. SO_2_ may disrupt gastrointestinal function (22) and subsequently impair immune system, thereby increasing susceptibility to pathogen infection and leading to OID, particularly under extreme meteorological conditions.

This study aimed to investigate the lagged and non-linear effects of SO_2_ on OID counts in Fuzhou, addressing a critical research gap. We employed multiple analytical approaches to assess the interaction between meteorological factors and SO_2_ on OID cases, thereby evaluating the effects of their joint exposure. Stratified analyses identified vulnerable subpopulations. These findings are crucial for developing effective preventive measures to reduce OID burden and offer a theoretical foundation for OID prevention and control in China.

## 2 Materials and methods

### 2.1 Study region and population

This study was conducted in Fuzhou, the capital of Fujian Province, a coastal city in southeastern China. OID consistently ranks among the top three Class C infectious diseases in Fujian, with an annual incidence of 88.3/100,000 in 2017 (23). Located between 25°15′-26°39′N and 118°08′-120°31′E, Fuzhou features a typical subtropical monsoon climate with abundant heat, humidity, and sunshine, especially in summer. By 2022, its permanent population reached 8.5 million, with an annual average temperature of 20.8 °C.

### 2.2 Data collection

OID case data were collected from the Infectious Disease Surveillance System from January 1, 2015, to December 31, 2019, covering Fuzhou residents. Patients were diagnosed based on official diagnostic criteria (Diagnostic criteria for infections diarrhea, WS 271–2007). According to China's regulations, all health institutions must report OID cases online within 24 h of diagnosis. Records included birth date, onset date, residence, gender, and age. Daily case counts were calculated based on the onset date and subsequently aggregated to construct a daily time-series dataset for further analysis. Daily meteorological data, including mean temperature (MT) and mean relative humidity (MRH), were obtained from the China Meteorological Data Network. Daily sulfur dioxide (SO_2_) data were obtained from the Nationwide Urban Air Quality Real-Time Monitoring Platform, averaged across five monitoring stations to represent citywide exposure levels.

### 2.3 Data analysis

This study is an ecological study, utilizing compiled time-series data for subsequent analyses. Firstly, we summarized the basic characteristics of OID counts and environmental variables. Secondly, we combined the generalized additive model (GAM) with the distributed lag non-linear model (DLNM) to investigate the relationship between the number of OID and MT, MRH, and SO_2_. Thirdly, we conducted subgroup analyses by gender and age groups. Fourthly, the model introduced the product terms of environmental variables to assess the potential interactive effects. Finally, the multiplicative scale, relative excess risk due to interaction (RERI) and attributable proportion (AP) were then used to evaluate the effects of interactions between meteorological factors and SO_2_ on OID (24).

#### 2.3.1 Distributed lag non-linear model

The quasi-Poisson regression combined with DLNM was utilized to assess the lagged and non-linear effects of environmental variables on OID counts, allowing for overdispersion in the daily number of OID cases. The combined model can be described by the following formula:


$$\begin{array}{l} Y_{t}\sim quasi-Poisson\left(u_{t}\right) \end{array}$$ (1)

$$\begin{array}{l} Log\left(u_{t}\right)=\alpha +cb\left(M_{t}\right)+ns\left(N_{t},df\right)\\ \;+ns\left(time,df\right)+ns\left(immune,df\right)+dow_{t}\\ \;+holiday_{t}+Lag\left(res,1\right)+Lag\left(res,2\right) \end{array}$$ (2)

Where *Y*_*t*_and *u*_*t*_ represent the observed and expected counts of OID on the day *t*, respectively, α denotes the intercept of the model. *cb*(.) represents the cross-basis matrix based on two natural cubic splines (*ns*) of environmental variables (*M*_*t*_) and lag days. The degrees of freedom (*df* ) for environmental variables and lag days were selected by the Quasi-Poisson Akaike Information Criterion (QAIC) (4). Considering the effects of the factors and the incubation period of OID, the lag days were set to 28 days for MT, 14 days for MRH, and 7 days for SO_2_ (4, 16). *N*_*t*_ are the other environmental variables than that considered in the cross-basis, which were controlled by natural cubic spline with 3 *df* . The long-term trend and seasonal variation in OID counts were adjusted annually using a natural cubic spline with 7 *df* . *Immune* represents the immune population for OID, defined as the cumulative number of cases in the 90 days prior to onset. It was controlled using a natural cubic spline with 3 *df* (25). The model also includes categorical confounders for day of the week (*dow*_*t*_) and public holidays (*holiday*_*t*_). *Lag(res,1)* and *Lag(res,2)* are the first- and second-order lagged model residual error, which were included in the model to control the temporal autocorrelation of infectious diseases cases (4).

Due to the non-linear associations, we calculated relative risk (RR) and 95% confidence intervals (CIs) of the 5th and 95th percentiles of exposure distribution referent to the median value consistent with previous studies (4). Cumulative RRs and 95% CIs were computed for MT (0–28 days), MRH (0–14 days), and SO_2_ (0–7 days) based on lag patterns. Extreme conditions (5th and 95th percentiles) were assessed for environmental variables. Stratified analyses identified vulnerable populations by gender and age groups (< 1, 1–2, 3–4, and ≥5), with subgroup-specific cumulative RRs and 95% CIs calculated. Statistical significance was defined as a 95% CI excluding 1.

#### 2.3.2 Interaction analysis

We incorporated a product term of one cross-basis matrix and one strata into the base model to consider the interactive effects between different environmental factors, which could consider non-linear association cumulated over lag period of one factor at different levels of another factor. For example, to estimate the interaction between MT and SO_2_ on OID, the base model included an interaction term of the cross-basis matrix of SO_2_ and a strata of MT. We divided the strata variable into low (< 33%), median (33%−67%) and high (>67%) categories to ensure adequate sample and statistical efficiency. The formula for the interaction is shown below.


$$\begin{array}{l} Log\left(u_{t}\right)=\alpha +factor\left(X\right)+cb\left(M_{t}\right)+factor{\left(X\right)}^{*}cb\left(M_{t}\right)\\ \;+ns\left(N_{t},df\right)+ns\left(time,df\right)+dow_{t}+holiday_{t}\\ \;+ns\left(immune,df\right)+Lag\left(res,1\right)+Lag\left(res,2\right) \end{array}$$ (3)

where *factor (X)* is the strata variables, factor (X)^*^ *cb (M*_*t*_*)* is the interaction term, the other terms are same as those in base model. The interaction analyses were also conducted for the MT and MRH, and MRH and SO_2_.

To analyze the multiplicative or additive interaction between environmental variables on OID counts, we categorized MT, MRH and SO_2_ into three levels (low, median and high) based on the 33rd and 67th percentiles. To capture the cumulative lag effects of the variables, 28-day moving averages for MT, 14-day moving averages for MRH, and 7-day moving averages for SO_2_ were calculated before generating the dummy variables. Dummy variables were created to represent the nine possible combinations of any two variables. For example, we defined the low-level SO_2_ and low-level MRH as the reference (RR_00_), median-level SO_2_ and low-level MRH as the RR_10_, high-level SO_2_ and low-level MRH as the RR_20_, low-level SO_2_ and median-level MRH as the RR_01_, low-level SO_2_ and high-level MRH as the RR_02_, median-level SO_2_ and median-level MRH as the RR_11_, median-level SO_2_ and high-level MRH as the RR_12_, high-level SO_2_ and median-level MRH as the RR_21_, high-level SO_2_ and high-level MRH as the RR_22_. Dummy variables were introduced into the quasi-Poisson model to examine the interaction effects. The formula of model is shown below.


$$\begin{array}{l} Y_{t}\sim quasi-Poisson\left(u_{t}\right) \end{array}$$ (4)

$$\begin{array}{l} Log\left(u_{t}\right)=\alpha +A+B+A^{*}B+ns\left(C_{t},df\right)\\ \;+ns\left(time,df\right)+ns\left(immune,df\right)+dow_{t}\\ +holiday_{t}+Lag\left(res,1\right)+Lag\left(res,2\right) \end{array}$$ (5)

Where *A* and *B* denote two environmental variables, and A^*^B is the interaction term. Both *A* and *B* are three-level categorical variables, resulting in four interaction product term parameters, which represent the multiplicative scale. *C*_*t*_ is another environmental variable that was not included in the interaction terms, which was controlled by natural cubic spline with 3 *df* .

The multiplicative scale was used to reflect potential multiplicative interactions, while RERI and AP were applied to assess potential additive interactions. The formulas for RERI and AP are shown below.


$$\begin{array}{l} \;RERI_{11}=RR_{11}-RR_{01}-RR_{10}+1\\ \;RERI_{21}=RR_{21}-RR_{01}-RR_{20}+1\\ \;RERI_{12}=RR_{12}-RR_{02}-RR_{10}+1\\ RERI_{22}=RR_{22}-RR_{02}-RR_{20}+1 \end{array}$$ (6)

$$\begin{array}{l} \;AP_{11}=\frac{RERI_{11}}{RR_{11}}\\ \;AP_{21}=\frac{RERI_{21}}{RR_{21}}\\ \;AP_{12}=\frac{RERI_{12}}{RR_{12}}\\ AP_{22}=\frac{RERI_{22}}{RR_{22}} \end{array}$$ (7)

When the multiplicative scale is greater than 1, it indicates a positive multiplicative interaction; when it is less than 1, it suggests a negative multiplicative interaction; and when it equals 1, no multiplicative interaction is present. If both RERI and AP are less than 0, it indicates an antagonistic effect between the two factors; conversely, if both are greater than 0, it suggests a synergistic effect. If either RERI or AP equals 0, it signifies that there is no additive interaction between the two factors. The 95% CI was estimated by the delta method, following previous studies (24).

#### 2.3.3 Sensitivity analysis

We conducted several sensitivity analyses to ensure the robustness of our results, following previous studies (4, 8). First, we varied the degrees of freedom (3–5) for the variables and lags in the cross-basis functions. Second, we adjusted the degrees of freedom for the temporal trend to 6–8. Third, we modified the degrees of freedom for the confounders to 4–6. Finally, we controlled for autocorrelation by using the first-order lagged variables of the logarithmic value of daily counts plus one [*Lag(log(Y*_*t*_+*1),1)*] instead of the residual error [*Lag(res,1)* + *Lag(res,2)*] (25). All statistical tests were performed at a significance level of 0.05 (two-tailed) using R version 4.3.3.

## 3 Result

Table 1 summarized the daily cases of OID by gender and age groups, and the basic characteristics of daily environmental factors. Table 1 showed that a total of 20,977 cases of OID were reported in Fuzhou City from 2015 to 2019. Among these cases, there were 12,490 males (59.54%) and 15,650 infants aged 2 years and below (74.61%). The median values of MT, MRH and SO_2_ were 21.5 °C, 75%, and 5.7 μg/m^3^. Table S1 showed that the incidence rate of OID in Fuzhou during the study period exhibited an increasing trend followed by a decline.

**Table 1:** Descriptive statistics of daily counts of OID and environmental variables during 2015–2019 in Fuzhou, China.

Variable	*n* (%)	Min	P05	P25	Median	P75	P95	Max
Total OID	20,977 (100%)	0	2	4	7	13	40	113
Male	12,490 (59.54%)	0	1	2	4	8	24	69
Female	8,487 (40.46%)	0	0	1	3	5	17	44
< 1	5,163 (24.61%)	0	0	1	2	4	9	34
1–2	10,487 (49.99%)	0	0	1	2	6	24	69
3–4	2,349 (11.20%)	0	0	0	1	2	5	22
5+	2,978 (14.20%)	0	0	0	1	2	4	37
MT (°C)		2.3	9.9	15.1	21.5	27.1	30.5	32.8
MRH (%)		33	54	66	75	83	94	99
SO_2_ (μg/m^3^)		2	3.7	4.6	5.7	7	9.4	17.9

OID, other infectious diarrhea; MT, mean temperature; MRH, mean relative humidity; SO_2_, sulfur dioxide; *n* (%), counts (percentage).

**Figure 1:**
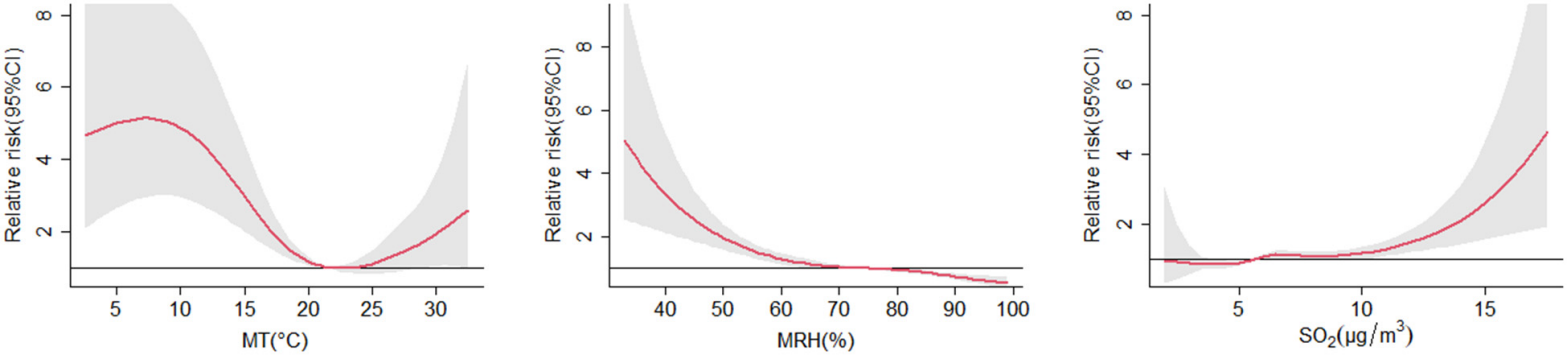
The associations between environmental variables and the counts of OID accumulate over lag days, referent at the median values. The reference values for daily MT, MRH, and SO_2_ are 21.5 °C, 75%, and 5.7 μg/m^3^, respectively. The 95% confidence intervals are represented by the shaded gray areas. OID, other infectious diarrhea; MT, mean temperature; MRH, mean relative humidity; SO_2_, sulfur dioxide.

Figure 1 depicted the cumulative exposure-response curves of each environmental variable over lag days. There were non-linear relationships between environmental factors and the counts of OID, with a U-shaped curve for MT, a L-shaped curve for MRH, and a J-shaped curve for SO_2_. The highest relative risk (RR) of MT was observed at 7.3 °C, reaching 5.14 (95% CI: 2.96, 8.91). The highest RR of MRH was observed at 33%, reaching 5.03 (95% CI: 2.56, 9.89). The highest RR of SO_2_ at the 17.9 μg/m^3^ was 5.13 (95% CI: 2.00, 13.19).

Table 2 described the cumulative relative risks of extreme MT, MRH, and SO_2_ on OID, stratified by gender and age groups. Significantly adverse RRs were observed for overall OID counts associated with extreme MT, MRH, and high SO_2_ concentrations. The harmful effects of extreme low MT were greater in individuals aged 3–4, while extreme high MT affected only the < 1 year age group. The effects of extreme MRH and SO_2_ were similar across age subgroups, with minimal differences between male and female groups. Additionally, subgroup analysis using the 1st and 99th percentiles for extreme levels yielded similar results (Table S2).

**Table 2:** The cumulative relative risks of OID for extreme MT, MRH, and SO_2_ over lag days, stratified by gender and age subgroups.

Environmental variable		Overall	Male	Female	< 1year	1–2 year	3–4 year	5+ year
MT	Cold (5th)	**4.90 (2.95, 8.12)**	**5.13 (2.90, 9.09)**	**5.50 (2.66, 11.35)**	**5.95 (2.63, 13.44)**	**3.89 (2.07, 7.32)**	**8.56 (2.79, 26.30)**	**7.00 (1.90, 25.70)**
	Hot (95th)	**2.05 (1.04, 4.04)**	**2.50 (1.14, 5.50)**	1.48 (0.58, 3.79)	**3.37 (1.30, 8.75)**	1.69 (0.58, 4.94)	1.11 (0.18, 6.79)	2.53 (0.73, 8.75)
MRH	Dry (5th)	**1.63 (1.40, 1.91)**	**1.66 (1.38, 1.99)**	**1.60 (1.29, 1.99)**	**1.45 (1.12, 1.88)**	**1.79 (1.48, 2.18)**	**1.94 (1.36, 2.76)**	1.44 (0.98, 2.12)
	Wet (95th)	**0.64 (0.53, 0.78)**	**0.66 (0.52, 0.83)**	**0.62 (0.47, 0.82)**	0.84 (0.61, 1.16)	**0.44 (0.34, 0.56)**	0.91 (0.57, 1.47)	1.21 (0.74, 1.99)
SO_2_	Low (5th)	0.85 (0.71, 1.02)	**0.72 (0.58, 0.89)**	1.06 (0.83, 1.37)	1.03 (0.76, 1.39)	0.92 (0.72, 1.16)	0.76 (0.49, 1.20)	0.72 (0.49, 1.05)
	High (95th)	**1.17 (1.01, 1.36)**	1.14 (0.95, 1.36)	1.19 (0.97, 1.46)	1.16 (0.89, 1.51)	**1.20 (1.00, 1.43)**	1.20 (0.85, 1.69)	0.78 (0.52, 1.17)

OID, Other Infectious Diarrhea; MT, Mean Temperature; MRH, Mean Relative Humidity; SO_2_, sulfur dioxide. Bold values indicate results that are statistically significant.

**Figure 2:**
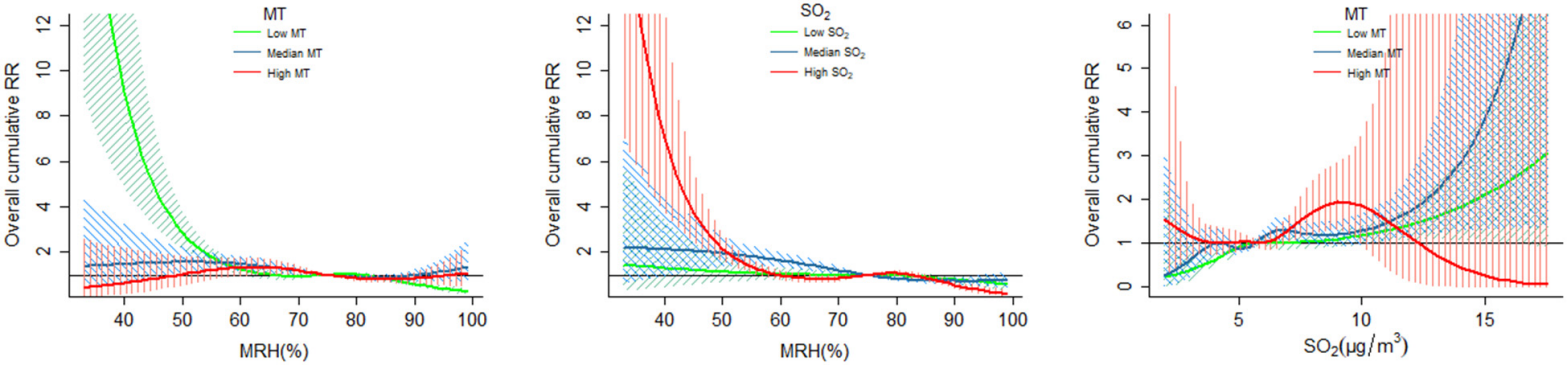
The interaction effects of MT, MRH, and SO_2_ on OID. OID, Other Infectious Diarrhea; MT, Mean Temperature; MRH, Mean Relative Humidity; SO_2_, sulfur dioxide; RR, Relative Risk; CI, Confidence Interval. The green, blue and red shaded areas represent the 95% CI for low, median, and high levels of the variables, respectively.

Figure 2, Supplementary Figure S1, Table 3 and Supplementary Table S3–5 illustrated the interactive effects of environmental variables on the risk of OID risk. We can visually observed that low level of MT and high level of SO_2_ amplified the adverse effect of low MRH on OID counts, and low and median levels of MT enhanced the adverse impact of high concentrations of SO_2_ on OID counts (Figure 2). For instance, the RRs of MRH (50%) on OID increased from the RR of 1.14 (95% CI: 0.72, 1.80) to 2.15 (95% CI: 1.65, 2.80) at low (< 33%) and high (>67%) levels of SO_2_, and decreased from 2.87 (95% CI: 2.20, 3.75) to 1.03 (95% CI: 0.58, 1.83) at low (< 33%) and high (>67%) levels of MT. Table 3 shows significant antagonistic effects between SO_2_ and MRH (RERI_22_ was −0.24 (95%CI: −0.42, −0.05) and AP_22_ was −0.27 (95%CI: −0.48, −0.05)), and between SO_2_ and MT (all the 95% CIs of RERI and AP < 0), but a synergistic interaction between MT and MRH (all the 95% CIs of RERI and AP >0). Table 3 also reveals a negative multiplicative interaction between SO_2_ and MRH, as well as between SO_2_ and MT (multiplicative scale less than 1). Conversely, a positive multiplicative interaction was observed between MT and MRH (multiplicative scale greater than 1).

**Table 3:** Multiplicative and additive interaction effects between environmental variables on OID risk.

Estimate	SO_2_ and MRH	SO_2_ and MT	MT and MRH
RR_00_	Ref	Ref	Ref
RR_10_	**1.15 (1.03, 1.29)**	**1.26 (1.15, 1.37)**	**0.67 (0.56, 0.79)**
RR_20_	**1.25 (1.11, 1.42)**	**1.42 (1.28, 1.57)**	**0.72 (0.57, 0.91)**
RR_01_	**0.87 (0.76, 0.99)**	0.88 (0.73, 1.06)	**0.79 (0.74, 0.84)**
RR_02_	**0.87 (0.77, 0.98)**	0.89 (0.70, 1.14)	**0.70 (0.64, 0.76)**
RR_11_	0.96 (0.86, 1.09)	0.91 (0.77, 1.08)	**0.59 (0.51, 0.70)**
RR_12_	0.93 (0.82, 1.06)	0.87 (0.68, 1.12)	**0.62 (0.53, 0.73)**
RR_21_	1.06 (0.94, 1.20)	0.86 (0.71, 1.04)	**0.68 (0.54, 0.87)**
RR_22_	0.89 (0.75, 1.05)	1.06 (0.83, 1.35)	**0.65 (0.52, 0.81)**
Multiplicative scale_11_	0.96 (0.83, 1.11)	**0.83 (0.71, 0.96)**	1.13 (0.98, 1.31)
Multiplicative scale_12_	0.93 (0.80, 1.07)	**0.78 (0.65, 0.94)**	**1.35 (1.15, 1.58)**
Multiplicative scale_21_	0.97 (0.84, 1.13)	**0.69 (0.57, 0.84)**	**1.20 (1.04, 1.38)**
Multiplicative scale_22_	**0.81 (0.68, 0.97)**	0.84 (0.68, 1.03)	**1.29 (1.08, 1.54)**
RERI_11_	−0.06 (−0.20, 0.09)	**−0.22 (−0.37**, **−0.07)**	**0.14 (0.04, 0.24)**
RERI_12_	−0.09 (−0.24, 0.06)	**−0.28 (−0.46**, **−0.09)**	**0.26 (0.16, 0.37)**
RERI_21_	−0.06 (−0.21, 0.09)	**−0.43 (−0.63**, **−0.23)**	**0.17 (0.07, 0.28)**
RERI_22_	**−0.24 (−0.42**, **−0.05)**	**−0.25 (−0.47**, **−0.03)**	**0.23 (0.11, 0.35)**
AP_11_	−0.06 (−0.21, 0.09)	**−0.24 (−0.41**, **−0.08)**	**0.23 (0.07, 0.40)**
AP_12_	−0.10 (−0.25, 0.06)	**−0.32 (−0.55**, **−0.08)**	**0.42 (0.25, 0.60)**
AP_21_	−0.06 (−0.20, 0.08)	**−0.50 (−0.76**, **−0.24)**	**0.25 (0.10, 0.41)**
AP_22_	**−0.27 (−0.48**, **−0.05)**	**−0.23 (−0.47, 0.00)**	**0.36 (0.15, 0.56)**

MT, Mean Temperature; MRH, Means Relative Humidity; SO_2_, sulfur dioxide; RR, Relative Risk; RERI, relative excess risk due to interaction; AP, proportion attributable. 0, 1 and, 2 represent the low, median and high level of environmental variables, respectively. Bold values indicate results that are statistically significant.

### 3.1 Sensitivity analysis

The results of sensitivity analysis indicated that the model results remain robust, with the alternative degrees of freedom for variables in the cross-basis function, the confounders, and the temporal trend. Additionally, the results changed slightly when using the first-order lagged variables of the logarithmic value of the daily counts plus one (Supplementary Figures S3–5).

## 4 Discussion

This study evaluated the independent effects of MT, MRH and SO_2_ on OID counts and quantified interaction effects using exposure-response relationships. Low MT, low MRH and high SO_2_ concentrations significantly increased OID risk. Notably, an antagonistic effect was observed between SO_2_ and MT, while MRH and MT showed a synergistic effect. Both multiplicative and additive interactions were confirmed. Additionally, children aged 3–4 were particularly vulnerable to extreme low MT, while the effects of extreme MRH and SO_2_ were consistent across age subgroups.

We found a U-shaped relationship between MT and OID counts, with both low and high MT increasing risk, though the effect of high MT was not statistically significant. Similar findings were reported in Jiangsu Province (26), low temperatures exhibited delayed and cumulative harmful effects. The increased risk of OID associated with low temperatures may be related to the pathogens that cause the disease. Cold environments enhance rotavirus survival and replication (27), though extremely cold days may reduce outdoor activity and subsequent exposure. Conversely, high temperatures correlated with increased OID cases (4), likely due to bacterial pathogens (such as Escherichia coli and Salmonella) growth, accelerated food spoilage, and changes in dietary habits, such as increased cold food consumption and reduced gastrointestinal defense (28).

This study found an L-shaped relationship between MRH and OID counts, highlighting the adverse effects of low humidity. This is consistent with a time-series study conducted in Guangzhou, China (8), where the risk of incidence decreased with increasing humidity. Study in Surabaya, India (9) also linked low humidity to higher infectious diarrhea risks. Laboratory evidence showed longer rotavirus survival at relative humidity ≤ 50%, with sustained infectivity (29). However, some studies reported a positive correlation between relative humidity and OID risk. For instance, Fang et al. (30) observed rising risks in Jiangsu Province when humidity ranged from 67 to 78%. These differences may stem from regional climatic variability and pathogen composition, underscoring the need for large-scale studies to validate these findings.

We found that high concentrations of atmospheric SO_2_ significantly increasing the incidence risk of OID. This aligns with previous studies reporting the harmful effects of SO_2_ on intestinal diseases (16) and inflammatory bowel disease (31). Although the mechanism remains unclear, one hypothesis is that SO_2_ may disrupt gut microbiota (22), impairing intestinal immunity and increasing susceptibility to infection and pathogen proliferation. Limited research exists on SO_2_ and infectious diarrhea. Ye and colleagues (18) found that air pollutants, including SO_2_, elevated rotavirus infection risk in children, supporting our findings. Further studies are needed to confirm these results.

Stratified analyses showed that individuals aged 3–4 were more susceptible to extreme low MT, while extreme MRH and SO_2_ had similar effects across different age groups. Previous studies (3, 32) have highlighted that individuals under 5 are particularly vulnerable to OID. We also observed negligible differences in OID effects between males and females. While a study in Tongcheng indicated that women are more vulnerable to OID (12), other studies (23, 32) reported that males are more susceptible. These discrepancies may be attributed to regional variations in pathogen composition. Due to inconsistent and limited evidence on subgroup effects, further large-scale studies are necessary to draw definitive conclusions.

This study demonstrated that MT and MRH had a synergistic effect on OID, with low MT and low MRH amplifying this effect. We also indicated that SO_2_ and MT had an antagonistic effect on OID, with high SO_2_ and low MT amplifying this effect. However, evidence of interaction effects between environmental variables, particularly for SO_2_, remains limited, warranting further research to explore these interactions, especially between SO_2_ and meteorological factors. Studies on bacterial dysentery (BD), a type of infectious diarrhea, have identified interactions among meteorological factors. For instance, research in Jilin Province, China (33), found significant interactions between temperature and humidity and between temperature and precipitation on BD incidence (*P* = 0.004, 0.002). Similarly, Chang et al. (34) suggested that relative humidity and precipitation enhance the effect of temperature on BD, indicating that warm and humid conditions promote BD incidence.

These findings move beyond simply identifying risk factors; they help define the specific environmental contexts and demographic subgroups where interventions would be most effective. For instance, public health authorities should implement early warning systems during weather forecasts predicting cold and dry conditions coupled with high air pollution (specifically SO_2_). During these critical periods, targeted interventions—such as issuing health alerts, enhancing hygiene education in kindergartens and households with young children, and ensuring access to safe drinking water—could be prioritized to reduce the OID burden among the most vulnerable subgroup.

This study has some strengths. Firstly, we have filled the gaps on the association between SO_2_ and OID counts and on its interaction with ambient temperature or humidity. These findings have significant public health implications reducing disease burden of OID. For instance, children aged 3–4 should avoid exposure to environments with low temperature, low humidity and high SO_2_. Secondly, the insights gained on the relationship between these factors are crucial for developing an OID incidence prediction and warning system. Lastly, the innovative approach of multiplying a cross-basis matrix with stratified terms helps explore exposure-response curves across multiple categories while capturing non-linear and lagged effects.

This study also has several limitations. Firstly, potential confounders such as education level, economic status and health condition were not adjusted for due to unavailable data, limiting further stratified analysis. Secondly, as an ecological study, it cannot determine the individual exposure levels. Thirdly, the case data lack laboratory test results, making it impossible to identify the specific pathogens causing OID. In addition, pathogen-spectrum heterogeneity may produce mixed effects on exposure–response curves and interaction outcomes; avoid attributing mechanistic explanations to a single pathogen. Lastly, the calculated RRs may be unstable due to the small sample size for extreme exposure, requiring cautious interpretation.

## 5 Conclusion

This study is the first to report adverse effect of sulfur dioxide on OID counts, and found significant independent effects of ambient temperature and relative humidity. Moreover, there were synergistic effect between MT and MRH and antagonistic effect between SO_2_ and MT. The effect of low MT on OID counts was higher at low MRH or high SO_2_. These findings suggest that public health policies should target critical periods characterized by concurrent low temperature, low humidity, and high SO_2_ levels—conditions under which the risk of OID is synergistically amplified—to implement targeted interventions for vulnerable populations, particularly children aged 3–4 years, thereby effectively reducing the disease burden.

## Data Availability

The datasets presented in this article are not readily available because the data of OID cases that support the findings of this study cannot be shared publicly because the data contain sensitive patient information, and sharing local sensitive contagious disease data publicly without license is illegal. Data are available from corresponding author for researchers who meet the criteria for access to confidential data. Requests to access the datasets should be directed to Xian-E Peng, peng123456@fjmu.edu.cn.
