# Supplementary material for: Unraveling complex interactions of meteorological factors and sulfur dioxide on other infectious diarrhea: evidence from a subtropical city
Source: Front Public Health. 2026 Apr 13;14:1706739. doi: 10.3389/fpubh.2026.1706739 (PMC13111384; doi:10.3389/fpubh.2026.1706739)
Supplement: Supplementary file 1 [file Supplementary_file_1.docx]

**Supplementary materials**


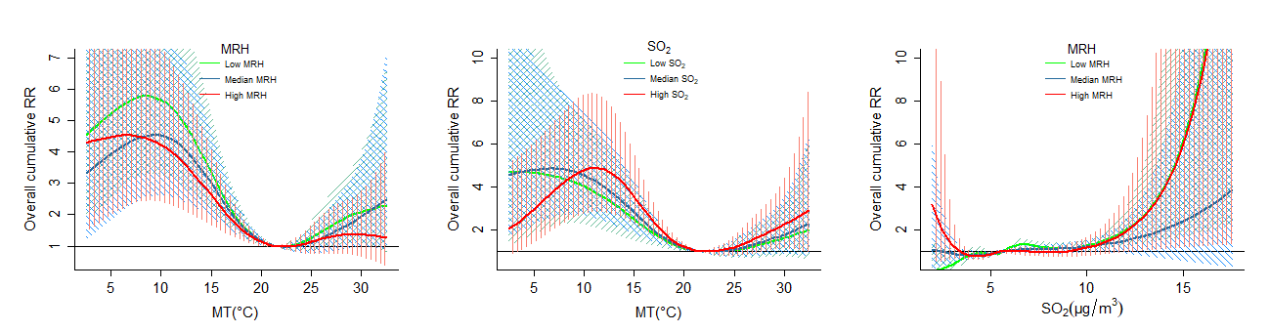


**Fig.S1.** The interaction effects of MT, MRH and SO_2_ on OID. OID stands for Other Infectious Diarrhea, MT represents Mean Temperature, MRH signifies Mean Relative Humidity, and SO_2_ means sulfur dioxide. RR refers to Relative Risk, CI indicates Confidence Interval. The green, blue and red shaded areas represent the 95% CI for low, median and high levels of the variables, respectively.


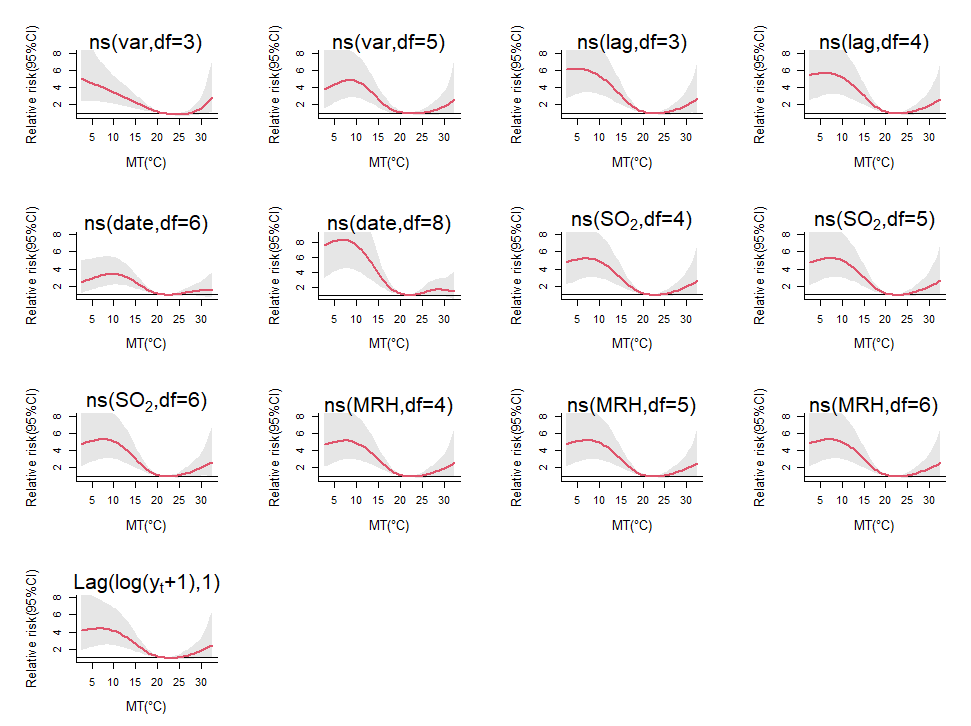


**Fig.S2.** Overall cumulative exposure-response curves between MT and OID incidence for sensitivity analysis, with a lag of 28 days, using the median value (21.5 °C) as reference. The 95% confidence intervals are represented by the shaded gray areas. OID refers to Other Infectious Diarrhea, MT stands for Mean Temperature, MRH represents Mean Relative Humidity, and SO_2_ means sulfur dioxide.


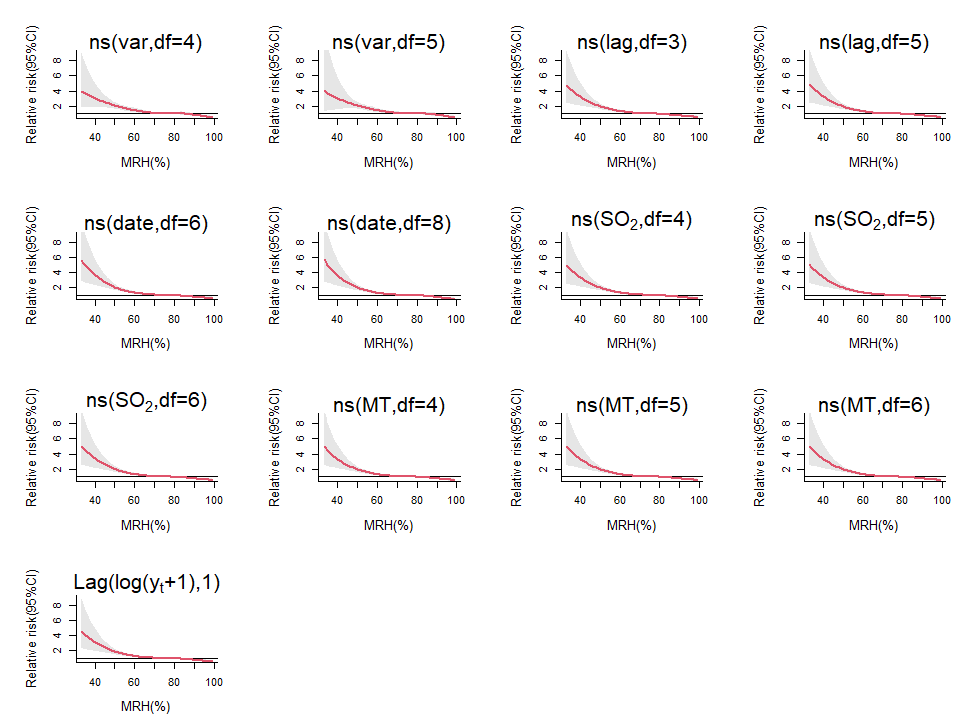


**Fig.S3.** Overall cumulative exposure-response curves between MRH and OID incidence for sensitivity analysis, with a lag of 14 days, using the median value (75%) as reference. The 95% confidence intervals are represented by the shaded gray areas. OID refers to Other Infectious Diarrhea, MT stands for Mean Temperature, MRH represents Mean Relative Humidity, and SO_2_ means sulfur dioxide.


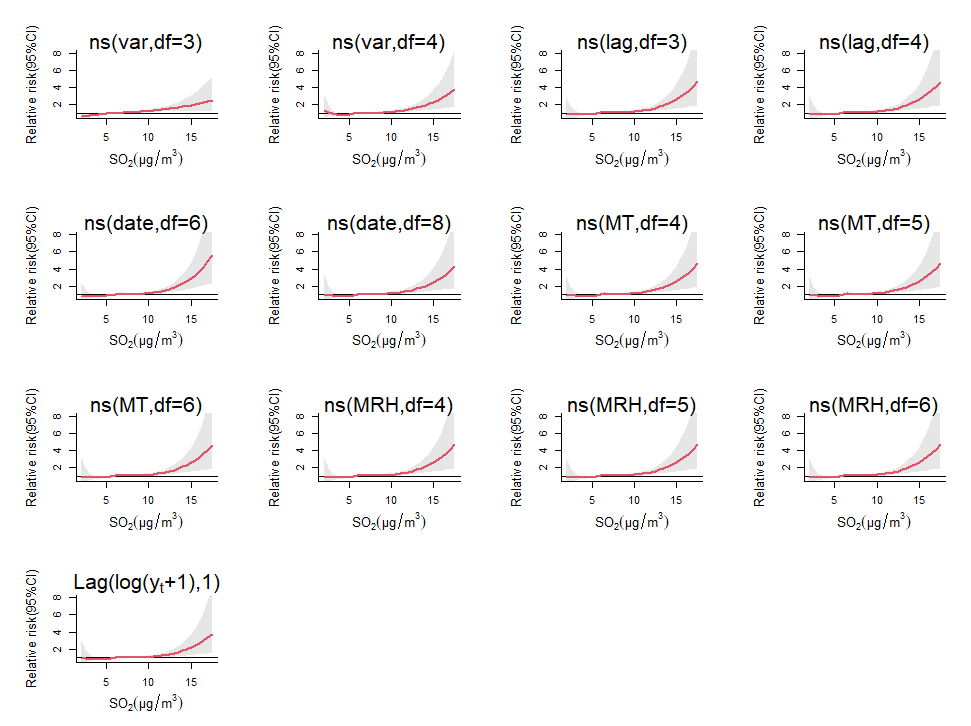


**Fig.S4.** Overall cumulative exposure-response curves between SO_2_ and OID incidence for sensitivity analysis, with a lag of 7 days, using the median value (5.7 μg/m^3^) as reference. The 95% confidence intervals are represented by the shaded gray areas. OID refers to Other Infectious Diarrhea, MT stands for Mean Temperature, MRH represents Mean Relative Humidity, and SO_2_ means sulfur dioxide.

**Table S1**

The annual counts and incidence rates of OID in Fuzhou during the study period.

| Variable | 2015 | 2016 | 2017 | 2018 | 2019 |
| --- | --- | --- | --- | --- | --- |
| count | 3312 | 3890 | 5916 | 4801 | 3058 |
| population (million) | 7.80 | 7.87 | 8.06 | 8.17 | 8.24 |
| incidence rate (per 100000 population) | 42.46 | 49.43 | 73.40 | 58.76 | 37.11 |

**Table S2**

The cumulative relative risks of OID for extreme MT, MRH and SO_2_ over lag days, stratified by gender and age subgroups.

| Environmental variable | | Overall | Male | Female | <1year | 1-2 year | 3-4 year | 5+ year |
| --- | --- | --- | --- | --- | --- | --- | --- | --- |
| MT | Cold(1th) | **5.14 (2.97,8.89)** | **5.02 (2.71,9.32)** | **6.60 (3.00,14.54)** | **6.98 (2.87,16.98)** | **3.91 (1.98,7.72)** | **11.15 (3.40,36.60)** | **6.55 (1.47,29.15)** |
|  | Hot(99th） | **2.28 (1.03,5.03)** | **2.63 (1.04,6.63)** | 1.80 (0.61,5.37) | **3.58 (1.18,10.85)** | 2.24 (0.64,7.92) | 1.26 (0.15,10.78) | 2.19 (0.53,9.08) |
| MRH | Dry(1th) | **2.54 (1.85,3.47)** | **2.61 (1.80,3.76)** | **2.48 (1.60,3.84)** | **1.78 (1.03,3.06)** | **3.07 (2.08,4.53)** | **2.81 (1.40,5.67)** | **2.38 (1.08,5.23)** |
|  | Wet(99th) | **0.55 (0.41,0.74)** | **0.57 (0.40,0.80)** | **0.52 (0.35,0.79)** | 0.83 (0.51,1.33) | **0.33 (0.23,0.47)** | 0.94 (0.46,1.91) | 1.24 (0.58,2.62) |
| SO_2_ | Low(1th) | 0.87 (0.63,1.21) | **0.66 (0.44,0.99)** | 1.23 (0.79,1.92) | 0.83 (0.49,1.40) | 1.19 (0.75,1.88) | 0.45 (0.18,1.15) | 0.62 (0.33,1.17) |
|  | High(99th) | **1.46 (1.18,1.80)** | **1.54 (1.20,1.97)** | **1.35 (1.01,1.81)** | **1.69 (1.18,2.41)** | **1.42 (1.11,1.82)** | 1.34 (0.82,2.20) | 1.16 (0.62,2.16) |

OID stands for Other Infectious Diarrhea, MT represents Mean Temperature, MRH signifies Mean Relative Humidity, and SO_2_ means sulfur dioxide. Bolded values indicate statistically significant results.

**Table S3**

The cumulative risk of OID associated with MRH under different levels of MT and SO_2_ (95% CI).

| Independent effect | | low-MT | median-MT | high-MT | low-SO2 | median-SO2 | high-SO2 |
| --- | --- | --- | --- | --- | --- | --- | --- |
| MRH(%) | |  |  |  |  |  |  |
| 40 | **3.35 (2.13,5.27)** | **9.05 (4.92,16.65)** | 1.48 (0.68,3.22) | 0.65 (0.19,2.18) | 1.30 (0.50,3.37) | 2.13 (0.99,4.60) | **7.02 (3.82,12.89)** |
| 50 | **1.96 (1.60,2.41)** | **2.87 (2.20,3.75)** | **1.57 (1.12,2.20)** | 1.03 (0.58,1.83) | 1.14 (0.72,1.80) | **1.96 (1.40,2.73)** | **2.15 (1.65,2.80)** |
| 60 | **1.30 (1.14,1.48)** | **1.27 (1.06,1.52)** | **1.49 (1.21,1.83)** | 1.32 (0.99,1.76) | 1.04 (0.82,1.32) | **1.65 (1.37,1.99)** | 0.99 (0.80,1.21) |
| 70 | 1.04 (0.96,1.12) | 0.95 (0.85,1.06) | **1.19 (1.05,1.34)** | **1.20 (1.02,1.40)** | 1.00 (0.88,1.14) | **1.22 (1.09,1.37)** | **0.85 (0.75,0.96)** |
| 80 | 0.96 (0.90,1.02) | 1.01 (0.93,1.11) | **0.89 (0.80,0.98)** | **0.85 (0.75,0.97)** | 0.97 (0.88,1.08) | **0.84 (0.77,0.93)** | 1.08 (0.98,1.19) |
| 90 | **0.74 (0.65,0.84)** | **0.61 (0.52,0.72)** | 0.98 (0.78,1.23) | 0.85 (0.65,1.13) | **0.80 (0.66,0.95)** | **0.74 (0.62,0.87)** | **0.57 (0.45,0.72)** |

The low, median and high categories were defined by the 33rd and 67th percentiles of the environmental variables. OID refers to Other Infectious Diarrhea, MT stands for Mean Temperature, MRH represents Mean Relative Humidity, and SO_2_ means sulfur dioxide. The 33rd and 67th percentile of MT are 17.2°C and 25.5 °C. The 33rd and 67th percentile of SO_2_ are 4.9 μg/m^3^ and 6.5 μg/m^3^. Bolded values indicate statistically significant results.

**Table S4**

The cumulative risk of OID associated with MT under different levels of MRH and SO_2_ (95% CI).

| Independent effect | | low-MRH | median-MRH | high-MRH | low-SO2 | median-SO2 | high-SO2 |
| --- | --- | --- | --- | --- | --- | --- | --- |
| MT(°C) | |  |  |  |  |  |  |
| 5 | **4.99 (2.63,9.47)** | **5.23 (2.61,10.48)** | **3.93 (1.87,8.24)** | **4.48 (2.01,10.00)** | **4.63 (1.84,11.66)** | **4.77 (2.28,9.98)** | **2.96 (1.44,6.07)** |
| 10 | **4.88 (2.94,8.08)** | **5.65 (3.29,9.71)** | **4.52 (2.63,7.77)** | **4.22 (2.43,7.32)** | **4.01 (2.27,7.09)** | **4.53 (2.67,7.66)** | **4.78 (2.76,8.27)** |
| 15 | **2.97 (2.00,4.42)** | **3.36 (2.18,5.16)** | **3.00 (1.92,4.67)** | **2.62 (1.69,4.07)** | **2.48 (1.52,4.06)** | **2.79 (1.82,4.28)** | **3.55 (2.30,5.46)** |
| 20 | **1.17 (1.06,1.29)** | **1.16 (1.05,1.29)** | **1.17 (1.05,1.31)** | **1.13 (1.02,1.27)** | **1.14 (1.02,1.28)** | **1.16 (1.05,1.29)** | **1.20 (1.08,1.34)** |
| 25 | 1.10 (0.81,1.48) | 1.27 (0.89,1.81) | 1.12 (0.80,1.57) | 1.13 (0.77,1.66) | 1.05 (0.71,1.55) | 1.08 (0.76,1.53) | 1.19 (0.82,1.72) |
| 30 | **1.94 (1.03,3.62)** | **2.09 (1.09,4.00)** | **1.94 (1.00,3.74)** | 1.37 (0.68,2.74) | 1.60 (0.79,3.26) | 1.77 (0.90,3.49) | **2.24 (1.15,4.36)** |

The low, median and high categories were defined by the 33rd and 67th percentiles of the environmental variables. OID refers to Other Infectious Diarrhea, MT stands for Mean Temperature, MRH represents Mean Relative Humidity, and SO_2_ means sulfur dioxide. The 33rd and 67th percentile of MRH are 69% and 80%. The 33rd and 67th percentile of SO_2_ are 4.9 μg/m^3^ and 6.5 μg/m^3^. Bolded values indicate statistically significant results.

**Table S5**

The cumulative risk of OID associated with SO_2_ under different levels of MRH and MT (95% CI).

| Independent effect | | low-MRH | median-MRH | high-MRH | low-MT | median-MT | high-MT |
| --- | --- | --- | --- | --- | --- | --- | --- |
| SO_2_(μg/m^3^) | |  |  |  |  |  |  |
| 4 | 0.84 (0.71,1.00) | 0.87 (0.64,1.18) | 0.80 (0.63,1.02) | **0.77 (0.61,0.97)** | 0.61 (0.48,0.77) | 0.98 (0.72,1.35) | 1.00 (0.72,1.39) |
| 6 | **1.07 (1.01,1.13)** | **1.17 (1.07,1.28)** | 1.04 (0.95,1.14) | 1.02 (0.92,1.13) | 1.00 (0.92,1.09) | **1.15 (1.04,1.27)** | 0.99 (0.86,1.15) |
| 8 | 1.08 (0.96,1.21) | 1.15 (0.94,1.40) | 1.09 (0.91,1.30) | 0.94 (0.76,1.17) | 1.03 (0.89,1.21) | 1.19 (0.97,1.47) | **1.72 (1.21,2.44)** |
| 10 | **1.17 (1.01,1.36)** | **1.26 (1.02,1.55)** | 1.21 (0.97,1.50) | 1.16 (0.81,1.65) | 1.17 (0.98,1.40) | 1.28 (0.99,1.66) | 1.84 (0.98,3.47) |
| 12 | **1.48 (1.19,1.84)** | **1.97 (1.30,2.97)** | 1.50 (0.91,2.49) | 1.91 (0.98,3.72) | **1.43 (1.09,1.86)** | **1.75 (1.18,2.60)** | 1.11 (0.12,10.60) |

The low, median and high categories were defined by the 33rd and 67th percentiles of the environmental variables. OID refers to Other Infectious Diarrhea, MT stands for Mean Temperature, MRH represents Mean Relative Humidity, and SO_2_ means sulfur dioxide. The 33rd and 67th percentile of MRH are 69% and 80%. The 33rd and 67th percentile of MT are 17.2 °C and 25.5 °C. Bolded values indicate statistically significant results.
